# Comparative mapping of chalkiness components in rice using five populations across two environments

**DOI:** 10.1186/1471-2156-15-49

**Published:** 2014-04-26

**Authors:** Bo Peng, Lingqiang Wang, Chuchuan Fan, Gonghao Jiang, Lijun Luo, Yibo Li, Yuqing He

**Affiliations:** 1 National Key Laboratory of Crop Genetic Improvement, National Center of Plant Gene Research and National Center of Crop Molecular Breeding, Huazhong Agricultural University, Wuhan 430070, China; 2 Shanghai Agrobiological Gene Center, Shanghai 201106, China

**Keywords:** *Oryza sativa* L., QTL, Rice, Chalkiness, Comparative mapping

## Abstract

**Background:**

Chalkiness is a major constraint in rice production because it is one of the key factors determining grain quality (appearance, processing, milling, storing, eating, and cooking quality) and price. Its reduction is a major goal, and the primary purpose of this study was to dissect the genetic basis of grain chalkiness. Using five populations across two environments, we also sought to determine how many quantitative trait loci (QTL) can be consistently detected. We obtained an integrated genetic map using the data from five mapping populations and further confirmed the reliability of the identified QTL.

**Results:**

A total of 79 QTL associated with six chalkiness traits (chalkiness rate, white core rate, white belly rate, chalkiness area, white core area, and white belly area) were mapped on 12 chromosomes using five populations (two doubled haploid lines and three recombinant inbred lines) across two environments (Hainan in 2004 and Wuhan in 2004). The final integrated map included 430 markers; 58.3% of the QTL clustered together (QTL clusters), 71.4% of the QTL clusters were identified in two or more populations, and 36.1% of the QTL were consistently detected in the two environments. The QTL could be detected again and showed dominance (*qWBR1*, *qWBR8*, *qWBR12*, and *qCR5*) or overdominance effects (*qWCR7*) for the rate of the white belly or white core, respectively, and all four QTL clusters derived from Zhenshan 97 controlling white belly rate were stably and reliably identified in an F_2_ population.

**Conclusions:**

Our results identified 79 QTL associated with six chalkiness traits using five populations across two environments and yielded an integrated genetic map, indicating most of the QTL clustered together and could be detected in different backgrounds. The identified QTL were stable and reliable in the F_2_ population, and they may facilitate our understanding of the QTL related to chalkiness traits in different populations and various environments, the relationships among the various chalkiness QTL, and the genetic basis for chalkiness. Thus, our results may be immediately used for map-based cloning of important QTL and in marker-assisted breeding to improve grain quality in rice breeding.

## Background

Rice (*Oryza sativa* L.) is one of the most important food crops worldwide and contributes to 40% of the total calorie intake for Chinese people. With the improvement of living standards, there is an increasing demand for better grain quality [1-3]. Rice grain quality is a complex character with several components, including grain appearance and milling, eating, cooking, and nutritional qualities; appearance quality is mostly determined by grain shape and endosperm opacity (or chalkiness) [3,4]. Chalkiness is divided into white belly, white core (WC), and white back (WB) in rice grain, depending upon its location on or within the endosperm. It represents a major problem in rice production in many rice-producing areas of the world because chalkiness results in inferior milling, cooking, eating, and nutritional quality [1,5-11]. Thus, grain chalkiness determines grain quality and price, and its reduction is an important goal of artificial breeding in rice.

Previous studies have shown that chalkiness is a complex quantitative trait that is controlled by polygenes and readily influenced by environmental factors [12-14]. During the last 15 years, many molecular marker-based QTL analyses of rice grain chalkiness have been conducted [10,11,15-23], and 82 QTL have been detected using numerous mapping populations (http://archive.gramene.org/qtl/). Therefore, great progress has already been made toward understanding the genetic basis of chalkiness; however, no gene controlling the trait has been cloned, and not much is known about the genetic mechanisms for crop genetic improvement.

Almost every previous experimental population for studying chalkiness was limited in size, restricted to a cross, or resulted in low-density maps, and most previous samples were planted in specific environments [15-19,24-29]. Two problems are apparent from previous studies. First, the QTL effects detected in a single population are extremely limited for detection of QTL with desirable power; accuracy depends on the genetic diversity in the parental lines, heritability of the traits, the size of the crosses, as well as the density of genetic markers [30]. Second, identifying the alignments and allelism of multiple QTL across populations is difficult because each study was based on different experimental populations, markers, data collection, and analytical methods, although comparative analysis has been confirmed as an effective way to identify multiple alleles and to collect a great quantity of research information from different studies [31,32]. In addition, little attention has been paid to the components of chalkiness, which have different genetic bases.

In this study, four populations derived from four crosses between a common female parent Zhenshan 97 (ZS97) and four male parents (H94, Delong 208 [DL208], Nanyangzhan [NYZ], and Wuyujing [WYJ]) with different degrees of chalkiness and diverse genetic backgrounds were used to detect QTL for chalkiness traits across two environments by using a series of simple sequence repeat (SSR) markers. Comparative mapping analysis was conducted by making use of another genetic population derived from ZS97 and Minghui 63 (MH63) in our laboratory [16,33]. Our experiment involved a systematic analysis of the genetic basis of grain chalkiness in rice. Additionally, we endeavored to confirm the genetic basis of the internal components of chalkiness to determine whether they have different behaviors in various genetic backgrounds and environments. Furthermore, to verify the reliability of QTL, we developed an F_2_ population derived from a cross between ZS97 (high-chalkiness rate) and WG97 (low-chalkiness rate). Thus, a systematic analysis of the genetic basis of grain chalkiness has paved the way for molecular marker-assisted selection and map-based cloning of important genes and QTL, as well as the genetic improvement of grain chalkiness and grain quality in rice and potentially other staple crops.

## Methods

### The mapping populations and the field experiment

The five mapping populations included two doubled haploid (DH) lines (ZS97/H94 and ZS97/WYJ) and three recombinant inbred lines (RILs; ZS97/DL208, ZS97/NYZ, and ZS97/MH63) that were derived from three crosses; ZS97 (*Oryza sativa* L. ssp. *indica*) was their common female parent [16,34-38]. An F_2_ population was derived from a cross between ZS97 and WG97 [39]. A total of six populations were used in this study.

The DH populations were derived from the cross of ZS97 with H94 (an *indica* variety with translucent endosperm) and WYJ (a *japonica* variety with a similar degree of chalkiness as ZS97) [35,38]. RIL population ZS97/DL208 was derived from a cross between ZS97 and DL208 (dull endosperm). ZS97/NYZ was derived from a cross between ZS97 and NYZ (a kind of *indica* red rice with greater chalkiness than ZS97), and the population ZS97/MH63 was derived from a cross between ZS97 and MH63 [16]. All populations and their parents were planted during the rice-growing season on the experimental farm of Huazhong Agricultural University in 2004 in Hainan and Wuhan, respectively. The day length in Hainan is longer than that in Wuhan (about 13 hours), while the temperature is much hotter in Wuhan (36–39°C) than that in Hainan (25–28°C).

An F_2_ population (396 individuals, then knocked-out a major QTL from 1398 individuals) was derived from a cross between ZS97 and WG97 (backcross female parent without chalkiness or with low-rate chalkiness, *O. sativa* L. ssp. *indica*) [39]. This F_2_ population and the parents were planted during the rice-growing season in 2008 on the experimental farm of Huazhong Agricultural University, Wuhan, China.

Each line was planted with two replications in each sowing, with each line containing 10 plants transplanted in a single row with 16.5-cm plant spacing and 26.4-cm row spacing. Field management essentially followed normal agricultural practice [35]. Field irrigation was maintained to avoid drought stress to the late-maturing lines.

### Traits

One hundred milled rice grains including broken grains were randomly selected from the middle six plants for each line and were put on a visualizer to identify those complying with the National Standard of the People’s Republic of China—Good Quality of Rice Grains (GB/T17891-1999). The grains with chalkiness were counted, and the percentage of chalky grains was calculated as the chalkiness rate (CR). For chalkiness area (CA), 20 grains with chalkiness were randomly selected, and the ratio of the CA to the whole kernel square for each grain was evaluated by visual assessment. Grains with WC and WB were further separated and counted. The parameters of white belly rate (WBR), white belly area (WBA), white core rate (WCR), and white core area (WCA) of WB and WC were estimated with the same method used for CR and CA. WCA and WBA could scarcely be distinguished from one another in ZS97 and NYZ because the areas were large and often fused together. All parameters for each sample of the lines and their parents were measured with two replications.

### DNA markers and assays

Polymorphic SSR markers involving all 12 chromosomes were detected for genotypes in the four populations: 218 for DH population ZS97/H94, 179 for RIL population ZS97/DL208, 190 for RIL population ZS97/NYZ, and 179 for RIL population ZS97/WYJ [36]. The primers of the RM series were designed based on previous studies [40,41], and those of the MRG series relied on the rice genome sequences of Monsanto Company [42]. The SSR assay was conducted as described previously [36].

### Data analysis

All genetic linkage maps were constructed by Mapmaker 3.0 [43]. The average of the measurements for each line in each population was utilized for QTL analysis. QTLMapper version 1.6 was based on a mixed linear model approach [44,45], and it was also employed to detect QTL containing the chalkiness traits of location-related chalkiness. In this analysis, likelihood ratio (LR) value *P* = 0.005 (equivalent to LOD = 4.03 for df = 6) was utilized as the threshold for claiming the presence of putative main QTL. The significance of the QTL effects was further tested by Bayesian analysis (*P* < 0.005). The peak points of the LR in the linkage map were taken as the putative positions of the effects. Dominant and overdominant effect analysis in the ZS97/WG97 population was taken as described previously [46]. The relative contribution of a genetic component was calculated as the proportion of phenotypic variance explained in the selected model.

### Integrated genetic map

An integrated genetic map was obtained using the JoinMap® 4.0 software program [47]. Information from the five mapping populations was integrated by the recombination frequencies and LOD values. Fixed order option was used to define the ordering of doubtful markers. The final integrated map included 430 markers.

## Results

### Measurements and segregations of the six chalkiness traits in four populations

Chalkiness traits of the parents (ZS97, H94, NYZ, and WYJ) and populations (ZS97/H94, ZS97/DL208, ZS97/NYZ, and ZS97/WYJ) in Hainan and Wuhan are shown in Table 1. There were no observations for the chalkiness traits in parent DL208 because all endosperms were opaque. The chalkiness traits in parent NYZ were observed in 100% of the samples and were much more prominent than those in other parents for CR, WCR, and WBR. The WCA and WBA were large and often overlapped; therefore, it was difficult to estimate size. There was a smaller amount of chalkiness area in H94, and the endosperms were relatively transparent; ZS97 and WYJ had a larger amount of chalkiness area, and grain WC was readily distinguishable from grain WB. In addition, the tremendous transgressive segregation phenomenon of traits was also observed in the four populations (Table 1). After the grain WC analysis of WB and other chalkiness-related traits, we found that the variation coefficient of CR and CA became greater than that before the traits’ subdivision (Table 1).

**Table 1:** Descriptive statistics of the traits in parents and the populations observed in Hainan and Wuhan

**Traits**^**a**^	**Local**^**b**^	**Parents**				**ZS97/H94 population**			**ZS97/DL208 population**			**ZS97/NYZ population**			**ZS97/WYJ population**		
		**ZS97**	**H94**	**NYZ**	**WYJ**	**Mean**	**Range**	**CV**^**c **^**%**	**Mean**	**Range**	**CV**^**c **^**%**	**Mean**	**Range**	**CV**^**c **^**%**	**Mean**	**Range**	**CV**^**c **^**%**
CR (%)	H	98.0	10.2	100.0	—	68.4	0.0-100.0	48.2	35.9	2.1-100.0	70.5	93.4	30.3-100.0	13.5	—	—	—
	W	94.5	6.3	100.0	85.5	44.4	2.0-100.0	76.8	23.8	0.0-90.0	78.7	94.5	31.7-100.0	11.3	83.2	10.0-100.0	27.4
CA (%)	H	30.3	17.5	75.3	—	28.3	0.0-71.5	52.1	22.5	5.5-66.0	47.3	37.7	8.8-75.6	37.4	—	—	—
	W	28.0	17.3	78.5	30.5	31.7	10.2-70.1	41.8	19.9	0.0-59.2	61.5	39.8	8.8-86.0	41.4	32.0	0.0-83.5	44.1
WCR (%)	H	14.0	6.0	100.0	—	8.8	0.0-100.0	238.8	24.3	0.0-100.0	76.5	58.7	0.0-100.0	68.7	—	—	—
	W	30.0	5.0	100.0	47.0	7.4	0.0-95.0	192.5	22.1	0.0-90.0	87.0	61.8	0.0-100.0	62.4	43.1	0.0-100.0	76.0
WBR (%)	H	97.0	4.0	100.0	—	59.9	0.0-100.0	66.3	12.1	0.0-93.7	170.3	64.4	0.0-100.0	64.0	—	—	—
	W	85.5	1.0	100.0	70.0	36.8	0.0-100.0	101.4	4.6	0.0-47.0	192.6	60.6	0.0-100.0	65.8	62.1	0.0-100.0	53.6
WCA (%)	H	15.0	15.0	—	—	11.1	0.0-80.0	184.4	23.2	0.0-52.5	45.0	—	—	—	—	—	—
	W	14.6	15.0	—	20.0	15.1	0.0-60.0	128.8	21.2	0.0-59.2	57.8	—	—	—	15.2	0.0-45.0	71.1
WBA (%)	H	18.0	10.0	—	—	19.2	0.0-65.0	74.1	3.9	0.0-23.5	141.6	—	—	—	—	—	—
	W	18.3	10.0	—	16.0	14.6	0.0-54.0	95.4	6.0	0.0-40.0	158.7	—	—	—	16.5	0.0-40.0	60.0

^a^CR, chalkiness rate; CA, chalkiness area; WCR, white core rate; WBR, white belly rate; WCA, white core area; WBA, white belly area.

^b^H, Hainan; W, Wuhan.

^c^CV, coefficient of variation.

### QTL analysis of the ZS97/H94 population

Six QTL were detected in population ZS97/H94, with three for CR and CA, respectively (Table 2). Two components traits (WC and WB) for chalkiness were further analyzed, and nine QTL (one for WCR, two for WCA, and three for WBR and WBA, respectively) were identified in this DH population (Table 2).

**Table 2:** QTL detected for chalkiness traits in population ZS97/H94

**Traits**^**a**^	**Chr**^**b**^	**Interval**	**QTL**	**Hainan**			**Wuhan**		
				**LOD**	**Add**^**c**^	**% Var**^**d**^	**LOD**	**Add**^**c**^	**% Var**^**d**^
CR									
	5	RM574-MRG0089	*qCR5-H+*	15.9	17.99	29.7	21	23.92	49.3
	6	RM435-RM170**( *wx *)**	*qCR6-H+*	14.6	16.47	24.9	3	6.7	3.9
	12	MRG2483-RM20A	*qCR12-H+*				2.7	6.56	3.7
CA									
	1	RM577-RM23	*qCA1-H+*				12.6	8.6	41.3
	6	RM170(*wx*)-RM589	*qCA6-H+*	4.9	4.96	11.3			
	9	RM278-RM553	*qCA9-H+*	8	6.98	22.4			
WCR									
	8	RM310-RM126	*qWCR8-H-*				5.3	-5.97	17.2
WBR									
	3	MRG2538-RM426	*qWBR3-H-*	2.3	-6.94	3.1			
	5	MRG0089-RM289	*qWBR5-H+*	12.3	20.62	27	11.1	20.93	31.5
	8	RM210-RM483	*qWBR8-H+*	11.5	17.71	19.9	5.2	14.49	15.1
WCA									
	8	RM483-RM339	*qWCA8-H-*				6	-6.43	10.9
	9	RM160-RM328	*qWCA9-H+*	8.4	9.07	21.6			
WBA									
	5	MRG0089-RM289	*qWBA5-H+*				4.3	4.04	8.4
	6	RM589-MX21 (*wx*)	*qWBA6-H+*	4.7	5.9	16.9			
	12	RM20A-RM179	*qWBA12-H+*				9.8	6.68	22.9

^a^CR, chalkiness rate; CA, chalkiness area; WCR, white core rate; WBR, white belly rate; WCA, white core area; WBA, white belly area.

^b^Chr, chromosome.

^c^The additive (Add) effects caused by QTL; the positive value indicates that the ZS97 allele increase the trait score, while the negative value indicates that the ZS97 allele decrease the trait score.

^d^The phenotypic variation (Var) explained by QTL.

Among the QTL for CR, *qCR5*-*H* had the largest effects on chromosome 5 and explained 29.7% of phenotypic variation in Hainan and 49.3% in Wuhan. *qCR5*-*H* was also detected as a major QTL for WBR in the two environments. *qCR6*-*H* in the interval of RM435-RM170 (*Wx*) on chromosome 6 was also detected as a major locus. It explained 24.9% of the phenotypic variation in Hainan and 3.9% in Wuhan; *qCR6*-*H* was also the locus for CA (*qCA6*-*H*) and WBA (*qWBA6*-*H*) in Hainan. Among the QTL for CA, *qCA1*-*H* was the largest, explaining 41.3% of phenotypic variation. After integrating the QTL, the total number of QTL decreased from 15 to 12 (Table 2).

The QTL sharing frequency were rather low in the two locations (Wuhan and Hainan). Only 4 out of 15 QTL were found in the two locations, and these were the CR or WB type. None of the QTL for CA and WCR were common to both locations at a high level (Table 2).

### QTL analysis of the ZS97/DL208 population

Fifteen QTL (two for CR, five each for WCR and WBA, and one each for CA, WBR, and WCA) controlling chalkiness traits were detected in population ZS97/DL308 (Table 3). *qCR9*-*D* had the largest effect for CR, explaining 24.2% of the phenotypic variation on chromosome 9, but it was only detected in Hainan. In the same locus, a QTL (*qWBR9*-*D*) was also detected for WBR in Wuhan. Both QTL could be integrated as one corresponding to WBR, with the negative additive effects of the allele from ZS97 in both environments (Hainan and Wuhan).

**Table 3:** QTL detected for chalkiness traits in population ZS97/DL208

**Trait**^**a**^	**Chr**^**b**^	**Interval**	**QTL**	**Hainan**			**Wuhan**			**Wuhan**^**e**^		
				**LOD**	**Add**^**c**^	**% Var**^**d**^	**LOD**	**Add**^**c**^	**% Var**^**d**^	**LOD**	**Add**^**c**^	**% Var**^**d**^
CR												
	5	MRG5972-RM480	*qCR5*-*D*+	3.2	11.07	16.3						
	9	RM159-RM524	*qCR9*-*D*-	2.9	-13.49	24.2						
CA												
	6	RM276-RM549	*qCA6*-*D*+	3.1	3.68	11.6						
WCR												
	1	MRG5464-MRG2148	*qWCR1*-*D*-				3.8	-6.67	11.8	2.6	-5.65	9.2
	3	RM203-RM422	*qWCR3*-*D*-				4.1	-6.46	11.1	2.7	-6.78	13.3
	6	MX21-RM585	*qWCR6*-*D*-	2.7	-19.08	99.2						
	7	RM445-RM418	*qWCR7*-*D*+				5.3	7.25	14.0	5.8	8.30	19.9
	12	RM235-RM17	*qWCR12*-*D*-							2.2	-5.35	8.3
WBR												
	9	RM159-RM524	*qWBR9*-*D*-	4.5	-6.72	36.4						
WCA												
	7	RM478-MRG4499	*qWCA7*-*D*+				2.7	4.49	15.4	2.0	4.84	19.0
WBA												
	3	RM251-RM282	*qWBA3*-*D*-	7.4	-3.08	29.6						
	5	RM39-RM164	*qWBA5*-*D*+	3.4	1.81	10.2						
	8	RM433-RM447	*qWBA8*-*D*+	4.9	2.44	18.5						
	11	RM286-RM20B	*qWBA11*-*D*+				2.2	3.48	12.6			
	12	RM235-RM17	*qWBA12*-*D*+	2.7	1.49	6.9						

^a^CR, chalkiness rate; CA, chalkiness area; WCR, white core rate; WBR, white belly rate; WCA, white core area; WBA, white belly area.

^b^Chr, chromosome.

^c^The additive effects caused by QTL; the positive value indicates that the Zhenshan 97 allele increase the trait score, while the negative value indicates that the Zhenshan 97 allele decrease the trait score.

^d^The phenotypic variation explained by QTL.

^e^Repeat of the previous year’s results in Wuhan.

In Hainan, 99.2% of the whole phenotypic variation of WCA could be explained by one predominant QTL (*qWCR6*-*D*). However, this predominant QTL was replaced by three other QTL in Wuhan. For WCA, only one QTL (*qWCA7*-*1D*) was found in Wuhan, whereas none was detected in Hainan. For WBA, four QTL were found in Hainan and explained 65.1% of the phenotypic variation, whereas only one was detected in Wuhan. After integrating the QTL data, only one QTL (*qCR9*-*D* on chromosome 9) was shared in the two locations.

### QTL analysis of the ZS97/NYZ population

The chalkiness traits including CR and CA were analyzed in the population ZS97/NYZ, and 10 QTL were detected, two for CR and eight for CA (Table 4). When the two component traits WC and WB were analyzed separately, 14 QTL for chalkiness traits were found: two for CR, eight for CA, one for WCR, and three for WBR (Table 4). Interestingly, two QTL for CR on chromosome 6 could be integrated as one since both QTL had the same SSR marker (RM541). *qCR6*-*N* was also found to correspond to the loci for CA and WCR (*qCA6*-*3N* and *qWCR6*-*N*).

**Table 4:** QTL detected for chalkiness traits in population ZS97/NYZ

**Trait**^**a**^	**Chr**^**b**^	**Interval**	**QTL**	**Hainan**			**Wuhan**		
				**LOD**	**Add**^**c**^	**% Var**^**d**^	**LOD**	**Add**^**c**^	**% Var**^**d**^
CR									
	2	RM183-RM526	*qCR2*-*N*-	3.5	-3.33	6.8			
	6	RM527- MRG2498	*qCR6*-*1N*-	5.6	-3.65	8.1	5.0	-3.91	13.4
CA									
	1	RM488-RM246	*qCA1*-*N*-				3.6	-3.58	4.6
	3	RM545-RM517	*qCA3*-*1N*-				5.6	-5.09	9.2
	3	RM468-RM570	*qCA3*-*2N*-				2.6	-2.95	3.1
	6	RM190-RM587	*qCA6*-*1N*-	1.9	-2.79	3.9			
	6	RM585-RM557	*qCA6*-*2N*-				6.9	-4.52	7.3
	6	MRG2498-RM454	*qCA6*-*3N*-	3.4	-3.77	7.1			
	9	RM296-RM321	*qCA7*-*N*-				4.8	-4.12	6.0
	11	RM332-RM167	*qCA8*-*N*-				9.1	-5.36	10.2
WCR									
	6	MRG2498-RM454	*qWCR6*-*N*-	2.9	-10.35	6.7			
WBR									
	1	RM490-RM600	*qWBR1*-*N*+	3.3	12.61	9.2			
	8	RM264-RM477	*qWBR8*-*N*+	3.0	11.05	7.1	5.0	12.32	9.4
	12	RM101-RM519	*qWBR12*-*N*+	3.6	14.55	12.3	3.3	12.40	9.6

^a^CR, chalkiness rate; CA, chalkiness area; WCR, white core rate; WBR, white belly rate; WCA, white core area; WBA, white belly area.

^b^Chr, chromosome.

^c^The additive effects caused by QTL; the positive value indicates that the Zhenshan 97 allele increase the trait score, while the negative value indicates that the Zhenshan 97 allele decrease the trait score.

^d^The phenotypic variation explained by QTL.

The frequency of the QTL sharing was low in the two locations in this population. Only three QTL were common to the two locations, two for WBR and one for CR. No QTL for CA was shared in the two locations at a high level, although eight QTL were detected for this trait.

### QTL analysis of the ZS97/WYJ population

Seven QTL including CR and CA were detected in population ZS97/WYJ, four for CR and three for CA (Table 5). When two component traits (WC and WB) for chalkiness were further analyzed, a total of 19 QTL were detected: seven for WCR, two for WCA, six for WBR, and four for WBA (Table 5).

**Table 5:** QTL detected for chalkiness traits in population ZS97/WYJ

**Trait**^**a**^	**Chr**^**b**^	**Interval**	**QTL**	**Wuhan**		
				**LOD**	**Add**^**c**^	**% Var**^**d**^
CR						
	2	RM263-RM221	qCR2-W-	3.3	-6.63	8.7
	4	RM335-MRG5943	qCR4-W-	3.9	-5.30	6.9
	7	RM82-RM125	qCR7-W-	3.4	-6.06	7.3
	9	RM296-RM285	qCR9-W+	3.8	6.28	7.8
CA						
	3	MRG2803-RM282	qCA3-W-	5.4	-4.15	8.6
	6	RM190-RM510	qCA6-W+	3.9	3.47	6.0
	9	RM285-MRG6094	qCA9-W+	3.6	3.20	5.1
WCR						
	3	RM36-MRG0002	qWCR3-1W-	9.0	-12.56	14.3
	3	RM130-RM570	qWCR3-2W-	3.1	-6.90	4.3
	4	RM335-MRG5943	qWCR4-1W-	13.8	-14.17	18.2
	4	RM142-RM177	qWCR4-2W+	3.2	8.09	6.3
	4	RM252-RM241	qWCR4-3W+	10.4	11.92	12.9
	8	RM80-RM149	qWCR8-W-	4.3	-9.29	8.3
	9	RM285-MRG6094	qWCR9-W+	8.6	12.40	13.9
WBR						
	1	RM84-RM283	qWBR1-1W-	4.1	-8.59	7.7
	1	RM129-RM9	qWBR1-2W+	4.2	8.08	6.8
	5	RM87-RM334	qWBR5-W+	2.5	6.09	3.9
	8	RM152-RM38	qWBR8-W-	13.3	-14.04	17.6
	9	RM296-RM285	qWBR9-W+	7.6	10.73	10.3
	11	RM536-RM287	qWBR11-W-	8.0	-10.29	9.4
WCA						
	1	RM259-RM312	qWCA1-W+	3.8	4.01	13.8
	4	RM335-MRG5943	qWCA4-W-	4.4	-3.88	12.8
WBA						
	1	RM84-RM283	qWBA1-W-	2.5	-2.64	7.1
	7	RM505-RM18	qWBA7-W-	2.0	-2.53	6.5
	8	RM210-RM80	qWBA8-W+	3.0	2.61	8.0
	11	RM536-RM287	qWBA11-W-	4.8	-3.95	15.9

^a^CR, chalkiness rate; CA, chalkiness area; WCR, white core rate; WBR, white belly rate; WCA, white core area; WBA, white belly area.

^b^Chr, chromosome.

^c^The additive effects caused by QTL; the positive value indicates that the ZS97 allele increase the trait score, while the negative value indicates that the ZS97 allele decrease the trait score.

^d^The phenotypic variation explained by QTL.

Typically multiple QTL controlled the chalkiness traits, and Table 5 shows that the alleles of positive or negative effects (increasing or decreasing trait value) were dispersed in the two parents, with positive alleles at one or more loci and negative alleles at another locus. These dispersed alleles showed an overdominant phenomenon in population ZS97/WYJ. Similar phenotypes could be found in the two parents, and many QTL were detected in one location. In QTL mapping, however, phenotypic variation of the two parents was not detected.

### The QTL comprehensive analysis using five populations

Seventy QTL controlling chalkiness traits (including CR, CA, WCR, WCA, WBR, and WBA) were detected on all 11 chromosomes (except chromosome 10) in the four populations (ZS97/NYZ, ZS97/DL208, ZS97/H94, ZS97/WYJ). In addition, nine QTL controlling chalkiness traits had previously been detected in the population ZS97/MH63 [16]. Therefore, there were 79 QTL affecting chalkiness in five populations with six traits distributed in 36 distinct locations. The comprehensive QTL in the five populations were analyzed by the order of the chromosomes in rice (Additional file 1: Table S1).

On chromosome 1, nine QTL were detected in the five populations, and five loci were residual after integrating the QTL. There were two QTL in the first locus located on the short arm of chromosome 1 in the interval of MRG5464-MRG2148 (population ZS97/DL208) and that of C161-R753 (population ZS97/MH63), respectively. Thus, overlapping QTL existed, and both of the QTL were detected in Wuhan. This locus controlled WCR in population ZS97/DL208, with WCR decreasing if the allele was derived from ZS97. However, this locus controlled CR in population ZS97/MH63. The second locus also had two QTL, and both QTL in the RM84-RM283 interval controlled WBR and WBA. The locus contained three overlapping QTL controlling WBR, CA, and WCA, respectively.

On chromosome 2, two overlapping QTL controlling CR were detected in population ZS97/NYZ and ZS97/WYJ, respectively. The alleles from ZS97 were associated with increased CR, and both of the QTL belonged to one locus.

On chromosome 3, eight QTL were detected in four populations (population ZS97/MH63 was not included). The eight QTL were interspersed at six loci, three of which contained two QTL. These two QTL were located in two overlapping intervals of RM251-RM282 and MRG2803-RM282 and controlled WBA and CA in populations ZS97/DL208 (Hainan) and ZS97/WYJ (Wuhan), respectively. There were also two QTL controlling CA and WCR, respectively, in the overlapping interval of RM468-RM570 and RM130-RM570 at the sixth loci detected in Wuhan; both QTL were also possible for two independent loci. Interestingly, the other four loci had only one QTL.

On chromosome 4, five QTL controlling WC and distributed over three loci were detected in population ZS97/WYJ. Three QTL controlling CR, WCR, and WCA, respectively, were interspersed in the RM335-MRG5943 interval, and the chalkiness effects decreased if the alleles were derived from ZS97. Single QTL controlling WCR were detected at the second and third locus, respectively, and the additive effects were detected if the allele was derived from ZS97.

On chromosome 5, ten QTL in four populations (except population ZS97/NYZ) were detected, and only one originated from population ZS97/MH63. At the first locus, six QTL controlling CR, WBR, and WBA were detected in the interval of RM574-MRG0089-RM289 (population ZS97/H94) or RG360-C734a (population ZS97/MH63). Interestingly, the allele derived from ZS97 increased WBR while decreasing WC, and this locus was previously shown to affect grain width and WBR in population ZS97/MH63 [16]. These vital effects were found in both Wuhan and Hainan, and this phenomenon was noteworthy. At the third locus, three overlapping QTL controlling CR or WBR were detected in three populations in both Hainan and Wuhan.

On chromosome 6, fourteen QTL interspersed at three loci were detected in five populations. Two types of QTL could be divided into eight QTL at the first locus (*Wx* locus): one type of QTL controlling WCR or CR was detected in both Wuhan and Hainan, and the other type of QTL controlling WBA and CA. The alleles from different populations had effects in various directions. Thus, there was no specific type of chalkiness traits because of the two types of QTL, although it was possible that two tightly linked loci existed. The *Alk* gene encoding soluble amylase (starch synthesis–related enzyme), another important gene for cooking and eating quality, was also at this locus (Additional file 1: Table S1).

**Figure 1:**
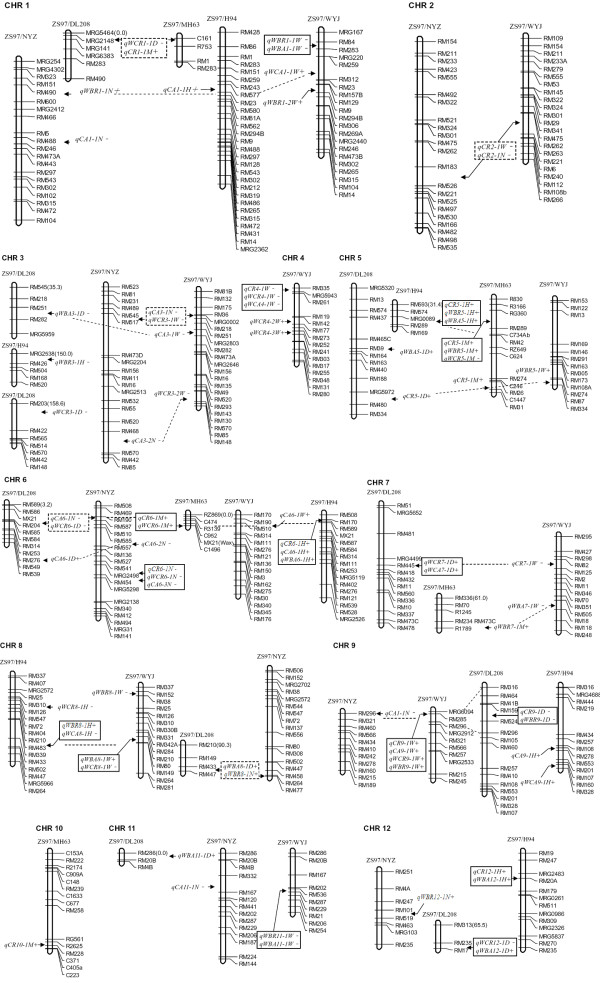
**The mapped locations of the integrated QTL profiles for the rice chalkiness traits in five populations.** The QTL clusters are indicated by the panes or dotted lines; further the lines and dotted panes indicate the QTL cluster emerged after combination, while the solid panes indicate that the QTL clusters existed before combination.

On chromosome 7, five QTL were detected at three loci in three populations (Figure 1). One QTL controlling CR was detected at the first locus, and two QTL controlling grain WC were detected at the second locus. Two QTL controlling grain WB were also detected at the third locus.

On chromosome 8, eight QTL were interspersed at four loci, and four QTL at the third loci were detected in four populations (except population ZS97/MH63). Interestingly, WBR increased and WCA decreased simultaneously if the alleles were derived from ZS97 in population ZS97/H94, while WBA increased and WCR decreased simultaneously if the alleles were derived from ZS97 in population ZS97/WYJ. All these QTL demonstrated reciprocal inhibition among different types of chalkiness traits, similar to a locus of QTL on chromosome 5. Moreover, two QTL detected at the fourth locus from ZS97 enhanced WBR or WBA.

On chromosome 9, nine QTL interspersed at three loci were detected in four populations (except population ZS97/MH63), and seven of them could be divided into two types controlling CA and WB at the first locus. Interestingly, multiple alleles were also detected, and this was similar to the *Wx* locus on chromosome 6. A QTL controlling CA or WCA was detected in the other two loci, respectively.

On chromosome 10, only one QTL controlling CR was detected [16].

On chromosome 11, four QTL were detected at three loci in population ZS97/WYJ, with two QTL at the third locus. Both WBR and WBA decreased simultaneously if the alleles were derived from ZS97.

On chromosome 12, five QTL were detected at two loci. Three QTL controlling CR, WBR, and WBA, respectively, were at the first locus. Two QTL at the second locus controlled increased WBA and decreased WCR simultaneously in population ZS97/DL208 if the alleles were derived from ZS97; this character was similar to the locus on chromosome 5 (Additional file 1: Table S1, Figure 1).

Taken together, some overlapping QTL controlled the same types of chalkiness in five populations. Therefore, we could integrate the 79 QTL into 36 loci, 21 of which clustered together and contained 64 QTL (Additional file 1: Table S2).

### Confirmation of the identified QTL

There were 79 QTL affecting chalkiness traits with six chalkiness traits (CR, CA, WCR, WCA, WBR, and WBA) across two environments. These QTL were integrated into 36 distinct locations on 12 chromosomes in five populations (Figure 1 and Additional file 1: Table S1). Twenty-one of the 36 distinct locations contained 64 QTL clustered together (QTL clusters), which were distributed on 11 chromosomes (except for chromosome 10) in the five populations. With regard to the rate of chalkiness traits, 11 of 21 QTL clusters from ZS97 displayed rate increases, while the others displayed rate decreases (Additional file 1: Table S3). Interestingly, 15 of 21 QTL clusters (71.4%) were identified in two or more populations (Additional file 1: Table S3), and the QTL cluster on chromosome 6 was detected in all five populations (Figure 1). Thus, the QTL clusters were relatively stable, and 13 of 36 distinct locations (36.1%) were consistently detected in Wuhan and Hainan (Additional file 1: Table S2).

To further confirm QTL, WG97 (ZS97 genetic background, with low or no chalkiness) and ZS97 were chosen as parents to construct an F_2_ population (1398 individuals) in 2008 in Wuhan. One main-effect QTL (*qCR5*-*H*+, the phenotypic variation explained by QTL for 49.3% and 29.7% in Wuhan and Hainan, respectively, Table 2) controlling CR, WBR, and WBA was knocked out by marker-assisted selection from the F_2_ population with two tightly linked molecular markers (RM574 and MRG0089). Consequently, 396 individuals derived from ZS97 were further analyzed by the tightly linked SSR markers (RM445-RM418, MRG5972-RM480, RM490-RM600, RM264-RM477, and RM101-RM519), respectively. The results indicated that the QTL effects could be reproduced and showed dominance (*qWBR1*, *qWBR8*, *qWBR12* and *qCR5*) or overdominance effects (*qWCR7*) for the rate of the chalkiness traits in this F_2_ population (ZS97/WG97). Moreover, five QTL could individually explain more than 10% of the variation of the trait; more than 15% of WBR and 23% of CR were explained if the gene regions were derived from ZS97 (Table 6). *qWBR1*, *qWBR8*, and *qWBR12* controlling WBR were identified again, and those were similar to the QTL clusters on chromosomes 1, 8, and 12, respectively (Table 6, Additional file 1: Table S3). Another QTL cluster controlling WBR was detected on chromosome 9 (Additional file 1: Table S3), and it was also found across eight environments [19]. Therefore, all four QTL clusters derived from ZS97 that controlled WB rate were stable and reliable (Additional file 1: Table S3).

**Table 6:** **Validation of 5 QTL in F**_**2 **_**population ZS97/WG97**

**Trait**^**a**^	**Chr**^**b**^	**Interval**	**QTL**	**Wuhan 2008**				**Hainan 2004**			**Wuhan 2004**			**Wuhan 2002**		
				**LOD**	**Add**^**c**^	**Dom**^**d**^	**% Var**^**e**^	**LOD**	**Add**^**c**^	**% Var**^**e**^	**LOD**	**Add**^**c**^	**% Var**^**e**^	**LOD**	**Add**^**c**^	**% Var**^**e**^
WBR	8	RM264-RM477	*qWBR8*	4.6	6.30	8.89	12.5	3.0	11.05	7.1	5.0	12.32	9.4			
WBR	12	RM101-RM519	*qWBR12*	7.7	10.53	11.53	16.1	3.6	14.55	12.3	3.3	12.40	9.6			
WCR	7	RM445-RM418	*qWCR7*	4.2	10.81	23.12	12.5				5.3	7.25	14.0	5.8	8.30	19.9
WBR	1	RM490-RM600	*qWBR1*	3.8	12.68	17.21	10.2	3.3	12.61	9.2						
CR	5	MRG5972-RM480	*qCR5*	7.7	12.18	14.68	16.4	3.2	11.07	16.3						

^a^WBR, white belly rate; CR, chalkiness rate.

^b^Chr, chromosome.

^c^The additive effects caused by QTL, the positive value indicates that the ZS97 allele increase the trait score.

^d^Dom, dominant effects.

^e^The phenotypic variation explained by QTL.

## Discussion

Comparative analysis of multiple QTL mapping by alignment to a common genetic map offers a more complete picture of the genetic control of a trait than can be obtained by any other approach [48]. In this study, 79 QTL controlling six chalkiness traits (CR, WCR, WBR, CA, WCA, and WBA) were detected in five populations (ZS97/H94, ZS97/NYZ, ZS97/DL208, ZS97/WUJ, and ZS97/MH63). The QTL were integrated into 36 loci, 21 of which were clustered together and contained 64 of the QTL. In addition, 15 of 21 (71.4%) QTL clusters were found in two or more populations (Additional file 1: Table S3), and 13 of 36 (36.1%) distinct locations were consistently detected in Wuhan and Hainan (Additional file 1: Table S2). Therefore, the QTL clusters controlling grain chalkiness are relatively stable. However, 15 QTL still occur separately on different chromosomes. Our results confirm that chalkiness traits are mainly controlled by some major QTL, and they are important to varying degrees in five populations with different genetic backgrounds. In fact, there are many QTL in four populations (ZS97/H94, ZS97/WYJ, ZS97/DL208, and ZS97/NYZ), and a new QTL (R2625-C223, on chromosome 10) increased through integration the results of population ZS97/MH63. Thus, by subdividing the chalkiness traits, it was possible to detect many more QTL and to determine that they have reciprocal conformity in multiple environments and populations with different genetic backgrounds.

### Chalkiness QTL common to the different environments

The three populations (ZS97/H94, ZS97/DL208, and ZS97/NYZ) were all planted in Wuhan and Hainan, which represented two different environments. These populations were concomitantly planted in the same fields in two environments, respectively, with consistent soil fertility and field management [26]. ZS97 was their common parent, thus our results have a strong basis for comparison and reliability because it has been shown that some characters can be affected by different planting years and environments [19]. Seventy-nine QTL controlling chalkiness traits were integrated into 36 distinct locations on 12 chromosomes, and 13 of the 36 (36.1%) distinct locations were consistently detected in Wuhan and Hainan (Additional file 1: Table S2, Figure 1). The degree of correlation between the shared extent and characters of the QTL was consistent in the two environments, with the extent of sharing based on population being ZS97/H94 > ZS97/NYZ > ZS97/DL208 (Additional file 1: Table S1). The correlation coefficients between WBR and CR were higher than for the other traits in the two environments (Additional file 1: Table S4). Hence, the QTL associated with these traits were detected in the environment more frequently than QTL associated with other chalkiness traits. The reactions of the different populations in the same environment are also extraordinarily different [39], and chalkiness traits are affected by the weather conditions especially during the grain filling period [24]. However, the QTL identified in one environment did not necessarily yield the same results in another environment, possibly due to experimental errors or different test thresholds.

### Chalkiness QTL shared by different populations

**Figure 2:**
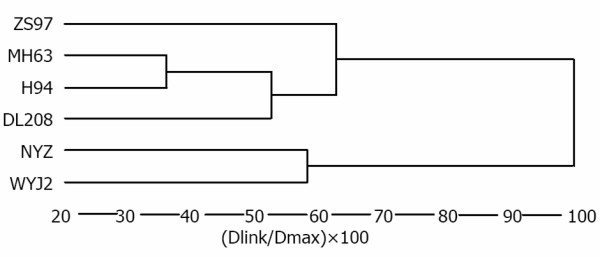
Cluster dendrogram of six parents used in this study as resolved by complete linkage using molecular marker difference as the distance measure.

After comparing the five populations in Wuhan and three populations in Hainan, we found few shared QTL for populations ZS97/H94, ZS97/NYZ, and ZS97/DL208, while populations ZS97/DL208, ZS97/WYJ, and ZS97/MH63 contained many of the same QTL. Fifteen of 21 (71.4%) QTL clusters were identified in two or more populations (Additional file 1: Table S3), and the QTL cluster on chromosome 6 was detected in five populations (Figure 1). Further research revealed that some QTL having minor effects in one population had major effects in other populations. On chromosome 1, one QTL controlling grain width was detected in population ZS97/DL208 and ZS97/MH63, and the effects were the same as chalkiness traits. Meanwhile, many QTL were mutual in population ZS97/H94 and ZS97/MH63, which were all low-chalkiness grains. Interestingly, our results are similar to earlier findings suggesting that the extent of QTL shared in populations is related to the genetic diversity of the different parents [15,49]; therefore, the six parents used in this were also analyzed (Figure 2).

### The relationship between chalkiness traits and the QTL

Through QTL analysis, the 79 QTL can be divided into three types. The first type has no specificity for chalkiness traits and only affects chalkiness such as the *Wx* locus on chromosome 6; this locus affects the different chalkiness types in various environments, populations, and even in the same population (Additional file 1: Table S1). The second type only has specificity for chalkiness traits without specificity for grain CR or CA. However, the third type controls only a single grain chalkiness trait and has the strongest specificity (Additional file 1: Table S1).

According to the relationship between chalkiness traits and the QTL, another three types are apparent in this study. The first type is “independent,” meaning that the QTL are independent from each other and a chalkiness trait is only affected by one specific QTL. The second type is “symbiosis,” which involves various chalkiness traits being controlled by a locus and showing an effect in the same direction. The third type is “competition” in which the QTL increases one chalkiness trait but simultaneously decreases other phenotypes (e.g., the QTL on chromosome 8) (Additional file 1: Table S1, Figure 1). Therefore, our results are similar to findings suggesting a complex relationship between chalkiness traits and the QTL. While chalkiness is under genetic control [16,50], the traits differ from each other with regard to their genetic mechanisms, but sometimes they also contact with each other [24]. Our results showed that the chalkiness traits were also under genetic control, the environmental impact was relatively weak, and dominance and overdominance effects were the key factors affecting grain chalkiness in rice.

### Benefits of multiple populations, multiple environments, and chalkiness traits

In this study, a large number of QTL controlling chalkiness traits in rice grain were detected, and the use of five populations, two environments, and six chalkiness traits will contribute to a more complete understanding of the genetic mechanism of the chalkiness traits. Moreover, the characterization of the QTL can be done more accurately if multiple environments and chalkiness traits are analyzed in multiple populations because of the low heritability of chalkiness traits [19,24,51]. Although some instrumentation exists for measuring grain chalkiness, grain WB cannot be distinguished from other chalkiness traits in this manner and the phenotypes could obscure the chalkiness QTL effects; therefore, we analyzed the chalkiness traits by visual inspection despite some difficulties. In addition, many QTL may be overlooked if the high-standard LOD value is adopted, while many more QTL may be discovered if the low-standard LOD value is used in analysis. Therefore, we used multiple populations, multiple environments, and chalkiness traits to prove the QTL reciprocally. Finally, multiple-population and multiple-environment analysis in our study is beneficial for investigating the different genetic mechanism of the chalkiness traits, the relationships among the chalkiness traits and the various manifestations in different environments, and populations with different genetic backgrounds. Interestingly, all of the previously reported loci were detected in our study except for a weak effect QTL (R2625-C223) located on chromosome 10 [16,19,50,52]. These results essentially satisfied our expectations that as many QTL as possible would be detected within multiple environments and for multiple traits; further, the results in one environment confirmed those in other environments. In addition, ZS97 is the common parent in five populations and is also a parent of Shanyou 63, which is the most widely grown rice hybrid in China. Consequently, our results have great significance for improving hybrid rice, which is widely planted in more than 20 countries around the world.

### The interpretation for chalkiness QTL and chalkiness formation mechanism

The chalkiness traits under genetic control involve many QTL [16,19,50,52]. These chalkiness traits are susceptible to environmental conditions and management practice under field conditions, and a considerable proportion of the traits are simultaneously caused by dominant or overdominant effects (Table 6). All the features considered in the current study commonly occurred in QTL analyses of yield traits in the literature [52,53]. Therefore, chalkiness trait occurrence is obviously a complex process, and it might be affected by the relationships among the source, storeroom, and streams [54]. Moreover, any change in these three factors or lack of coordination among them would influence or engender chalkiness in the endosperm. The modes of action and approaches for QTL were analyzed based on speculations about the three chalkiness traits. The utilization of QTL was a powerful means to estimate gene action of QTL and to conduct fine mapping of QTL to dissect complex rice endosperm chalkiness [55]. Our research also showed that some of the genes related to starch synthesis, such as *Wx* and *Alk*, might play an important role in the formation of chalkiness. In addition, various types of chalkiness QTL might be related to different chalkiness structure characteristics, filling periods, filling temperatures, and filling dynamics; for example, alpha-amylase has functional roles in the development of a chalky endosperm [56].

Since the chalkiness QTL effects can obscure each other’s phenotype, one main-effect QTL (*qCR5*-*H*+) controlling CR, WBR, and WBA was knocked out by marker-assisted selection from an F_2_ population. Our results showed that all four QTL clusters derived from ZS97 controlling the rate of chalkiness can be stably and reliably detected (Additional file 1: Table S3), and the other QTL are more critical in heterozygous individuals than the loci derived from ZS97 and show transgressive segregation in the F_2_ population (Additional file 1: Table S3). In addition, it was previously reported that the QTL on chromosome 5 controlling grain WB also controlled grain width, which is closely associated with chalkiness [16,19]. Moreover, the *flo4* gene controlling WC endosperm on chromosome 5 was also detected in the MRG5972-RM480 interval in this study [57]. For that reason, the effects of QTL in our comparative map are stable, although the other QTL need further confirmation. In rice breeding, some QTL derived from the alleles (non-ZS97), such as *qWBR3*-*H* (Table 2) and *qWCR6*-*D* (Table 3), decrease chalkiness traits. In theory, if all of these QTL were polymerized together for ZS97, grains (chalkiness-free) could be produced in diverse environments. Compared with the previous QTL, our results showed that the QTL clusters derived from ZS97 are stably and reliably identified in different populations and environments (Additional file 1: Table S3). Our comparison map includes all previous QTL of chalkiness traits, and most of the QTL in this study are unified and integrated.

## Conclusions

Our study identified 79 QTL associated with six chalkiness traits using five populations (two DH lines and three RILs) across two environments and obtained an integrated genetic map, which included 430 markers. The QTL were distributed across 36 distinct locations, and 58.3% of locations clustered together. In addition, 71.4% of the QTL clusters were identified in two or more populations, and 36.1% of QTL were consistently detected in two different environments. Results from this study facilitate our understanding of the QTL related to chalkiness traits in different populations and various environments and the genetic basis. Interestingly, the QTL could be detected in different populations, and four QTL clusters derived from ZS97 controlling WBR were stably and reliably detected in the F_2_ population, indicating that the identified QTL clusters are consistent in different populations and environments. Thus, our results have paved the way for rice breeding by marker-assisted selection and map-based cloning of important QTL or genes, as well as the genetic improvement of grain quality in rice and potentially other staple crops.

## Abbreviations

QTL: Quantitative trait loci; DH: Doubled haploid; RILs: Recombinant inbred lines; DNA: Deoxyribonucleic acid; ZS97: Zhenshan 97; DL208: Delong 208; NYZ: Nanyangzhan; WYJ: Wuyujing; MH63: Minghui 63; CR: Chalkiness rate; CA: Chalkiness area; WBR: White belly rate; WBA: White belly area; WCR: White core rate; WCA: White core area; SSR: Simple sequence repeat; LR: Likelihood ratio; WB: White core; WC: White belly; H: Hainan; W: Wuhan.

## Competing interests

The authors declare they have no competing interests.

## Authors’ contributions

BP and LQW were involved in the conception of the experiment, analysis, interpretation of the data, and drafting the article. CCF, GHJ, LJL, and YBL contributed to the interpretation of the results and helped to draft the manuscript. YQH designed the experiment, did quality control for the data, and performed the analysis. All authors read, contributed, and approved the manuscript.

## Supplementary Material

Additional file 1: Table S1 Integration of the QTL for chalkiness traits in five populations. **Table S2** Number of QTL for chalkiness traits in five populations. **Table S3** Information of the QTL clusters for the chalkiness traits in five populations. **Table S4** Coefficients of pairwise correlations of the same chalkiness trait between two environments in three populations. Click here for file
