# Supplementary material for: Comparative mapping of chalkiness components in rice using five populations across two environments
Source: BMC Genet. 2014 Apr 26;15:49. doi: 10.1186/1471-2156-15-49 (PMC4021085; doi:10.1186/1471-2156-15-49)
Supplement: Additional file 1: Table S1 — Integration of the QTL for chalkiness traits in five populations. Table S2 Number of QTL for chalkiness traits in five populations. Table S3 Information of the QTL clusters for the chalkiness traits in five populations. Table S4 Coefficients of pairwise correlations of the same chalkiness trait between two environments in three populations. [file 1471-2156-15-49-S1.docx]

**Comparative mapping of chalkiness components in rice using five populations across two environments**

Bo Peng^1^, Lingqiang Wang^1^, Chuchuan Fan^1^,Gonghao Jiang^1^, Lijun Luo^2^, Yibo Li^1^ and Yuqing He^1^^﹡^

^1^National Key Laboratory of Crop Genetic Improvement, National Center of Plant Gene Research and National Center of Crop Molecular Breeding, Huazhong Agricultural University, Wuhan 430070, China

^2^Shanghai Agrobiological Gene Center, Shanghai 201106, China

^﹡^Correspondence: [yqhe@mail.hzau.edu.cn](mailto:yqhe@mail.hzau.edu.cn)

^1^National Key Laboratory of Crop Genetic Improvement, Huazhong Agricultural University, Wuhan 430070, China

Tel: +86-027-87281689; Fax: 86-027-87287092

http://ibi.hzau.edu.cn/clstest/researchers.php?id=236

**Supplementary tables**

**Table S1 Integration of the QTL for chalkiness traits in five populations**

| **Chr** | **Order** | **Traits** |  | **QTL information^a^** | | | **Location^b^** | |  | **Population** | | **Method^c^** | **Grain traits QTL^d^** |
| --- | --- | --- | --- | --- | --- | --- | --- | --- | --- | --- | --- | --- | --- |
|  |  |  |  | **Interval** | **LOD** | **Allele** | **Hainan** | **Wuhan** |  | **Name** | **Type** |  |  |
| 1 | 1 | White core | rate | MRG5464-MRG2148 | 3.8 | - | no | Yes | D | | RIL | CIM | GT- |
|  |  | Chalkiness | rate | C161-R753 | 2.6 | + | / | yes |  | M | F2:3 | IM | TGW+, GW+ |
|  | 2 | White belly | rate | RM84-RM283 | 4.1 | - | / | yes |  | W | DH | CIM | TGW- |
|  |  | White belly | area | RM84-RM283 | 2.5 | - | / | yes |  | W | DH | CIM | TGW- |
|  | 3 | White belly | rate | RM490-RM600 | 3.3 | + | Yes | no |  | N | RIL | CIM | GW+ |
|  |  | Chalkiness | area | RM577-RM23 | 12.6 | + | No | yes |  | H | DH | CIM |  |
|  |  | White core | area | RM259-RM312 | 3.8 | + | / | yes |  | W | DH | CIM |  |
|  | 4 | White belly | rate | RM129-RM9 | 4.2 | + | / | yes |  | W | DH | CIM | GT- |
|  | 5 | Chalkiness | area | RM488-RM246 | 3.6 | - | no | yes |  | N | RIL | CIM |  |
| 2 | 1 | Chalkiness | rate | RM183-RM526 | 3.5 | - | yes | no |  | N | RIL | CIM | GW- |
|  |  | Chalkiness | rate | RM263-RM221 | 3.3 | - | / | yes |  | W | DH | CIM | TGW- |
| 3 | 1 | Chalkiness | area | RM545-RM517 | 5.6 | - | no | yes |  | N | RIL | CIM | GT- |
|  |  | White core | rate | RM36-MRG0002 | 9.0 | - | / | yes |  | W | DH | CIM | TGW-, GW-, GT- |
|  | 2 | White belly | area | RM251-RM282 | 7.4 | - | yes | no |  | D | RIL | CIM | GW+ |
|  |  | Chalkiness | area | MRG2803-RM282 | 5.4 | - | / | yes |  | W | DH | CIM | GW- |
|  | 4 | White belly | rate | MRG2538-RM426 | 2.3 | - | yes | no |  | H | DH | CIM | GW+ |
|  | 5 | White core | rate | RM203-RM422 | 4.1 | - | no | yes |  | D | RIL | CIM | GL- |
|  | 6 | Chalkiness | area | RM468-RM570 | 2.6 | - | no | yes |  | N | RIL | CIM | TGW-, GL-, GT- |
|  |  | White core | rate | RM130-RM570 | 3.1 | - | / | yes |  | W | DH | CIM |  |
| 4 | 1 | Chalkiness | rate | RM335-MRG5943 | 3.9 | - | / | yes |  | W | DH | CIM |  |
|  |  | White core | rate | RM335-MRG5943 | 13.8 | - | / | yes |  | W | DH | CIM |  |
|  |  | White core | area | RM335-MRG5943 | 4.4 | - | / | yes |  | W | DH | CIM |  |
|  | 2 | White core | rate | RM142-RM177 | 3.2 | + | / | yes |  | W | DH | CIM | GT- |
|  | 3 | White core | rate | RM252-RM241 | 10.4 | + | / | yes |  | W | DH | CIM | GT- |
| 5 | 1 | White belly | rate | MRG0089-RM289 | 12.3 | + | yes | yes |  | H | DH | CIM | GW+, GT+ |
|  |  | Chalkiness | rate | RM574-MRG0089 | 15.9 | + | yes | yes |  | H | DH | CIM | GW+, GT+ |
|  |  | White belly | area | MRG0089-RM289 | 4.3 | + | no | yes |  | H | DH | CIM | GW+, GT+ |
|  |  | Chalkiness | rate | RG360-C734a | 29.3 | + | / | yes |  | M | F2:3 | IM | TGW+, GW+, GT+ |
|  |  | White belly | rate | RG360-C734a | 35.2 | + | / | yes |  | M | RIL | IM | TGW+, GW+, GT+ |
|  |  | White core | rate | RG360-C734a | 4.5 | - | / | yes |  | M | RIL | IM | TGW+, GW+, GT+ |
|  | 2 | White belly | area | RM39-RM164 | 3.4 | + | yes | no |  | D | RIL | CIM |  |
|  | 3 | Chalkiness | rate | MRG5972-RM480 | 3.2 | + | yes | no |  | D | RIL | CIM |  |
|  |  | White belly | rate | RM87-RM334 | 2.5 | + | / | yes |  | W | DH | CIM |  |
|  |  | Chalkiness | rate | RG528-C1447 | 5.8 | + | / | yes |  | M | F2:3 | IM | GL- |
| 6 | 1 | Chalkiness | rate | RM435-RM170 (*wx*) | 14.6 | + | yes | yes |  | H | DH | CIM |  |
|  |  | White core | rate | MX21 (*wx*)-RM585 | 2.7 | - | yes | no |  | D | RIL | CIM | TGW+ |
|  |  | White core | rate | *wx*-R1952 | 4 | + | / | yes |  | M | RIL | IM | TGW+, GL+, GW+, GT+ |
|  |  | Chalkiness | rate | RG1952-C226 | 2.5 | + | / | yes |  | M | F2:3 | IM | TGW+, GL+, GW+, GT+ |
|  | 2 | Chalkiness | area | RM170 (*wx*)-RM589 | 4.9 | + | yes | no |  | H | DH | CIM |  |
|  |  | Chalkiness | area | RM190 (*wx*)-RM587 | 1.9 | - | yes | no |  | N | RIL | CIM |  |
|  |  | Chalkiness | area | RM190 (*wx*)-RM510 | 3.9 | + | / | yes |  | W | DH | CIM |  |
|  |  | White belly | area | RM589-MX21 (*wx*) | 4.7 | + | yes | no |  | H | DH | CIM |  |
|  | 3 | Chalkiness | area | RM585-RM557 | 6.9 | - | no | yes |  | N | RIL | CIM | GW- |
|  |  | Chalkiness | area | RM276-RM549 | 3.1 | + | yes | no |  | D | RIL | CIM |  |
|  | 4 | Chalkiness | rate | RM527-MRG2498 | 5.6 | - | yes | no |  | N | RIL | CIM | GL- |
|  |  | White core | rate | MRG2498-RM454 | 2.9 | - | yes | no |  | N | RIL | CIM | GL- |
|  |  | Chalkiness | area | MRG2498-RM454 | 3.4 | - | yes | no |  | N | RIL | CIM | GL- |
| 7 | 1 | Chalkiness | rate | RM82-RM125 | 3.4 | - | / | yes |  | W | DH | CIM |  |
|  |  | White core | area | RM478-MRG4499 | 2.7 | + | no | yes |  | D | RIL | CIM |  |
|  |  | White core | rate | RM445-RM418 | 5.3 | + | no | yes |  | D | RIL | CIM |  |
|  | 2 | White belly | area | RM505-RM18 | 1.8 | - | / | yes |  | W | DH | CIM |  |
|  |  | White belly | rate | R1245-R1789 | 2.7 | + | / | yes |  | M | RIL | IM |  |
| 8 | 1 | White belly | rate | RM152-RM38 | 13.3 | - | / | yes |  | W | DH | CIM |  |
|  | 2 | White core | rate | RM310-RM126 | 5.3 | - | no | yes |  | H | DH | CIM |  |
|  | 3 | White belly | rate | RM210-RM483 | 11.5 | + | yes | yes |  | H | DH | CIM |  |
|  |  | White core | area | RM483-RM339 | 6.0 | - | no | yes |  | H | DH | CIM |  |
|  |  | White belly | area | RM210-RM80 | 3.0 | + | / | yes |  | W | DH | CIM | GW+, GT+ |
|  |  | White core | rate | RM80-RM149 | 4.3 | - | / | yes |  | W | DH | CIM |  |
|  | 4 | White belly | area | RM433-RM447 | 4.9 | + | yes | no |  | D | RIL | CIM |  |
|  |  | White belly | rate | RM264-RM477 | 3.0 | + | yes | yes |  | N | RIL | CIM |  |
| 9 | 1 | Chalkiness | area | MRG6094-RM285 | 3.6 | + | / | yes |  | W | DH | CIM |  |
|  |  | Chalkiness | area | RM296-RM321 | 4.8 | - | no | yes |  | N | RIL | CIM | TGW-, GT- |
|  |  | Chalkiness | rate | RM159-RM524 | 2.9 | - | yes | no |  | D | RIL | CIM |  |
|  |  | White belly | rate | RM159-RM524 | 4.5 | - | no | yes |  | D | RIL | CIM |  |
|  |  | Chalkiness | rate | RM285-RM296 | 3.8 | + | / | yes |  | W | DH | CIM |  |
|  |  | White belly | rate | RM285-RM296 | 7.6 | + | / | yes |  | W | DH | CIM |  |
|  |  | White core | rate | MRG6094-RM285 | 8.6 | + | / | yes |  | W | DH | CIM |  |
|  | 2 | Chalkiness | area | RM278-RM553 | 8.0 | + | yes | no |  | H | DH | CIM |  |
|  | 3 | White core | area | RM160-RM328 | 8.4 | + | yes | no |  | H | DH | CIM |  |
| 10 | 1 | Chalkiness | rate | R2625-C223 | 2.5 | + | / | yes |  | M | F2:3 | IM | GT+ |
| 11 | 1 | White belly | area | RM286-RM20B | 2.2 | + | no | yes |  | D | RIL | CIM |  |
|  | 2 | Chalkiness | area | RM332-RM167 | 9.1 | - | no | yes |  | N | RIL | CIM |  |
|  | 3 | White belly | rate | RM536-RM287 | 8.0 | - | / | yes |  | W | DH | CIM |  |
|  |  | White belly | area | RM536-RM287 | 4.8 | - | / | yes |  | W | DH | CIM |  |
| 12 | 1 | Chalkiness | rate | MRG2483-RM20A | 2.7 | + | no | yes |  | H | DH | CIM | GW+ (Wuhan) |
|  |  | White belly | area | RM20A-RM179 | 9.8 | + | no | yes |  | H | DH | CIM | GW+ (Wuhan) |
|  | 2 | White belly | rate | RM101-RM519 | 3.6 | + | yes | yes |  | N | RIL | CIM |  |
|  | 3 | White core | rate | RM235-RM17 | 2.2 | - | no | yes |  | D | RIL | CIM |  |
|  |  | White belly | area | RM235-RM17 | 2.7 | + | yes | no |  | D | RIL | CIM |  |

^a^The “+” and “-” under allele column indicate the Zhenshan 97B allele effects.

^b^The “yes”, “no” and “/” indicate that the QTL was “detected”, “not detected” and “not investigated” under the location column, respectively.

^c^“IM” and “CIM” indicate that the methods used were “Interval mapping” and “composiion interval mapping”, respectively.

^d^The QTL for other grain traits associated TGW, GL, GW, GS and GT were also detected in the locations of the QTL for chalkiness-related traits, the “+” and “-” indicate the Zhenshan 97B allele effects.

**Table S2 Number of QTL for chalkiness traits in five populations**

| **Chr** | **QTL clusters identified**  **across traits^a^** | | | | **QTL clusters identified**  **across populations^b^** | | | | **Single**  **QTL** | **QTL common in two locations** |
| --- | --- | --- | --- | --- | --- | --- | --- | --- | --- | --- |
|  | **1T** | **2T** | **3T** | **4T** | **1P** | **2P** | **3P** | **5P** |  |  |
| 1 |  | 3 |  |  | 1 | 1 | 1 |  | 2 | 1 |
| 2 | 1 |  |  |  |  | 1 |  |  |  | 1 |
| 3 |  | 3 |  |  |  | 3 |  |  | 2 | 1 |
| 4 |  |  | 1 |  | 1 |  |  |  | 2 |  |
| 5 |  | 1 |  | 1 |  | 1 | 1 |  | 1 | 2 |
| 6 | 1 |  | 1 | 1 | 1 | 1 |  | 1 |  | 3 |
| 7 |  | 1 | 1 |  |  | 2 |  |  |  |  |
| 8 |  | 1 |  | 1 |  | 2 |  |  | 2 | 2 |
| 9 |  |  |  | 1 |  |  | 1 |  | 2 | 1 |
| 10 |  |  |  |  |  |  |  |  | 1 |  |
| 11 |  | 1 |  |  | 1 |  |  |  | 2 |  |
| 12 |  | 2 |  |  | 2 |  |  |  | 1 | 2 |
| Total | 2 | 12 | 3 | 4 | 6 | 11 | 3 | 1 | 15 | 13 |

^a^1T, 2T, 3T and 4T indicate the QTL clusters affecting one, two, three and four kinds of traits, respectively.

^b^1P, 2P, 3P and 5P indicate the QTL clusters were identified by one, two, three, and five populations, respectively.

**Table S3 Information of the QTL clusters for the chalkiness traits in five populations**

| Order | QTL clusters | Chr | Rate^a^ | | | Area^a^ | | | Populations |
| --- | --- | --- | --- | --- | --- | --- | --- | --- | --- |
|  |  |  | CR | WCR | WBR | CA | WCA | WBA |  |
| 1 | *qWBR1-1; qWBA1-1* | 1 |  |  | - |  |  | - | WYJ |
| 2 | *qWCR1-2; qCR1-2* | 1 | - | + |  |  |  |  | DL208; MH63 |
| 3 | *qCR2-1; qCR2-2* | 2 | + |  |  |  |  |  | WYJ; NYZ |
| 4 | *qCA3-1; qWCR3-1* | 3 | + |  |  | + |  |  | WYJ; NYZ |
| 5 | *qCR4-1; qWCR4-1; qWCA4-1* | 4 | - | - |  |  | - |  | WYJ |
| 6 | *qCR5-1; qWBR5-1; qWBA5-1* | 5 | + |  | + |  |  | + | H94; MH63 |
| 7 | *qCR5-1; qWBR5-1; qWCR5-1* | 5 | + | - | + |  |  |  | H94; MH63 |
| 8 | *qCR5-1; qWBR5-1* | 5 | - |  | - |  |  |  | DL208; WYJ; MH63 |
| 9 | *qCR6-1;WCR6-1* | 6 | + | + |  |  |  |  | NYZ; MH63 |
| 10 | *qCR6-1;qCA6-1;qWBA6-1* | 6 | + |  |  | + |  | + | WYJ; H94 |
| 11 | *qCR6-1;WCR6-1;qCA6-3* | 6 | - | - |  | - |  |  | DL208; NYZ |
| 12 | *qCA6-1; qWCR6-1* | 6 |  | - |  | + |  |  | DL208; NYZ |
| 13 | *WCR7-1;qWCA7-1* | 7 |  | + |  | + |  |  | DL208; WYJ |
| 14 | *qWBR8-1;qWCA8-1* | 8 |  |  | + |  | - |  | WYJ; H94 |
| 15 | *qWBA8-1;qWCR8-1* | 8 |  | - |  |  |  | + | NYZ; WYJ |
| 16 | *qWBA8-1; qWBR8-1* | 8 |  |  | - |  |  | - | DL208; NYZ |
| 17 | *qCR9-1;qCA9-1;qWBR5-1;qWCR5-1* | 9 | + | + | + | + |  |  | WYJ; NYZ |
| 18 | *qCR9-1;qWBR9-1* | 9 | - |  | - |  |  |  | DL208 |
| 19 | *qWBR11-1;qWBA11-1* | 11 |  |  | - |  |  | - | WYJ |
| 20 | *qWCR12-1;qWBA12-1* | 12 |  | - |  |  |  | + | DL208 |
| 21 | *qCR12-1;qWBA12-1* | 12 | + |  |  |  |  | + | H94 |

^a^The positive value indicates that the ZS97 allele increase the trait score, while the negative value indicates that the ZS97 allele decrease the trait score.

**Table S4 Coefficients of pairwise correlations of the same chalkiness trait between two environments in three populations**

| Populations | CR | CA | WCR | WBR | WCA | WBA |
| --- | --- | --- | --- | --- | --- | --- |
| ZS97/H94 | 0.71** | 0.36** | 0.46** | 0.77** | 0.26* | 0.40** |
| ZS97/DL208 | 0.43** | 0.35** | 0.34** | 0.57** | 0.08 | 0.27* |
| ZS97/NYZ | 0.89** | 0.79** | 0.88** | 0.92** | / | / |

*, ** Significance at *P* < 0.05 and *P* < 0.01, respectively.
